# Supplementary material for: Chemical inhibition of a CBASS anti-bacteriophage defense
Source: iScience. 2026 Jun 1;29(6):116174. doi: 10.1016/j.isci.2026.116174 (PMC13253105; doi:10.1016/j.isci.2026.116174)
Supplement: Document S1. Figures S1–S13 and Tables S1 and S2 [file mmc1.pdf]

iScience, Volume *29*

Supplemental information

Chemical inhibition of a CBASS anti-bacteriophage defense

Chengqian Zhang (张乘乾), Olga Rechkoblit, Shelley A.H. Dixon, Steven P. Angus, and Joseph P. Gerdt

Supplementary Information for

Chemical inhibition of a CBASS anti-bacteriophage defense

Chengqian Zhang (张乘乾)[1], Olga Rechkoblit[2], Shelley A. H. Dixon[3], Steven P. Angus[3], Joseph P. Gerdt[1]

[1] Department of Chemistry, Indiana University, Bloomington, IN 47405, USA.

[2] Department of Pharmacological Sciences, Icahn School of Medicine at Mount Sinai, New York, NY 10029, USA.

[3] Department of Pediatrics, Herman B. Wells Center for Pediatric Research, Indiana University School of Medicine, Indianapolis, IN 46202, USA.

Corresponding author: Joseph P. Gerdt (jpgerdt@iu.edu)

Document contains:

Supplementary Figures S1–S13

Supplementary Tables S1 & S2

SUPPLEMENTARY FIGURES

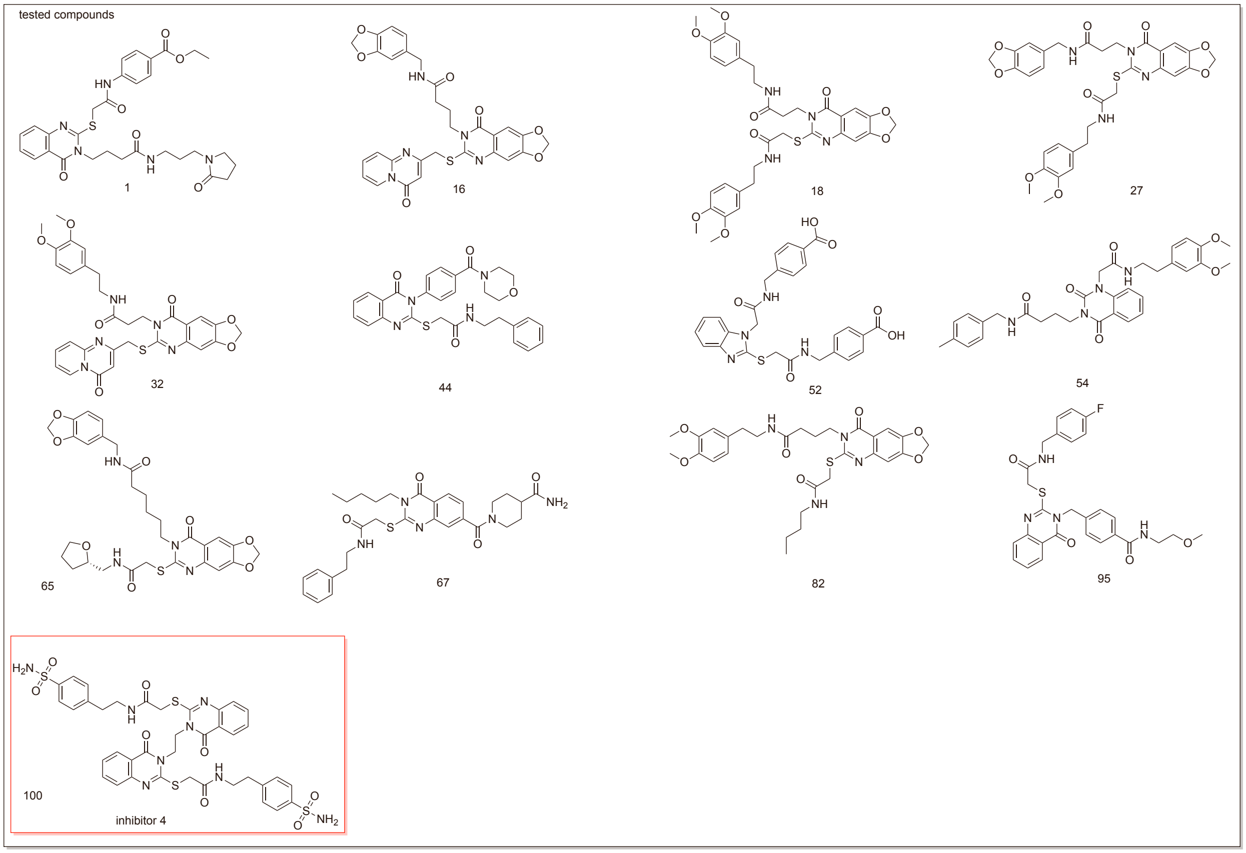

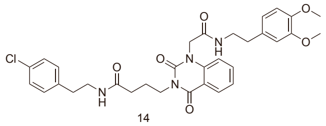

14

Figure S1. Group A of the top screening results. The boxed ones were purchased for inhibitor screening, the highlighted ones were validated as inhibitors. The docking score rank of each is noted with a number (see Table S2).

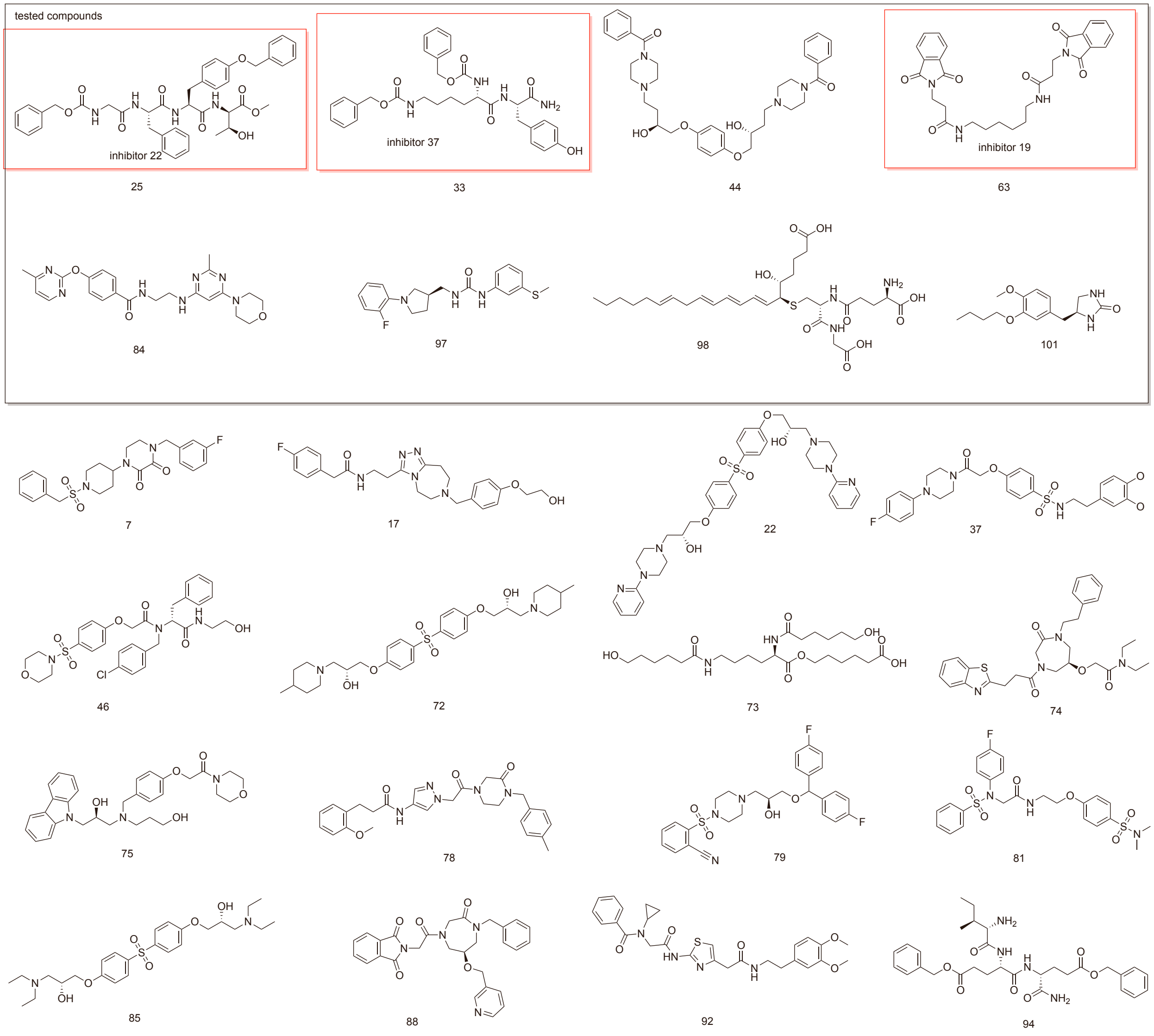

Figure S2. Group B of the top screening results. The boxed ones were purchased for inhibitor screening, the highlighted ones were validated as inhibitors. The docking score rank of each is noted with a number (see Table S2).

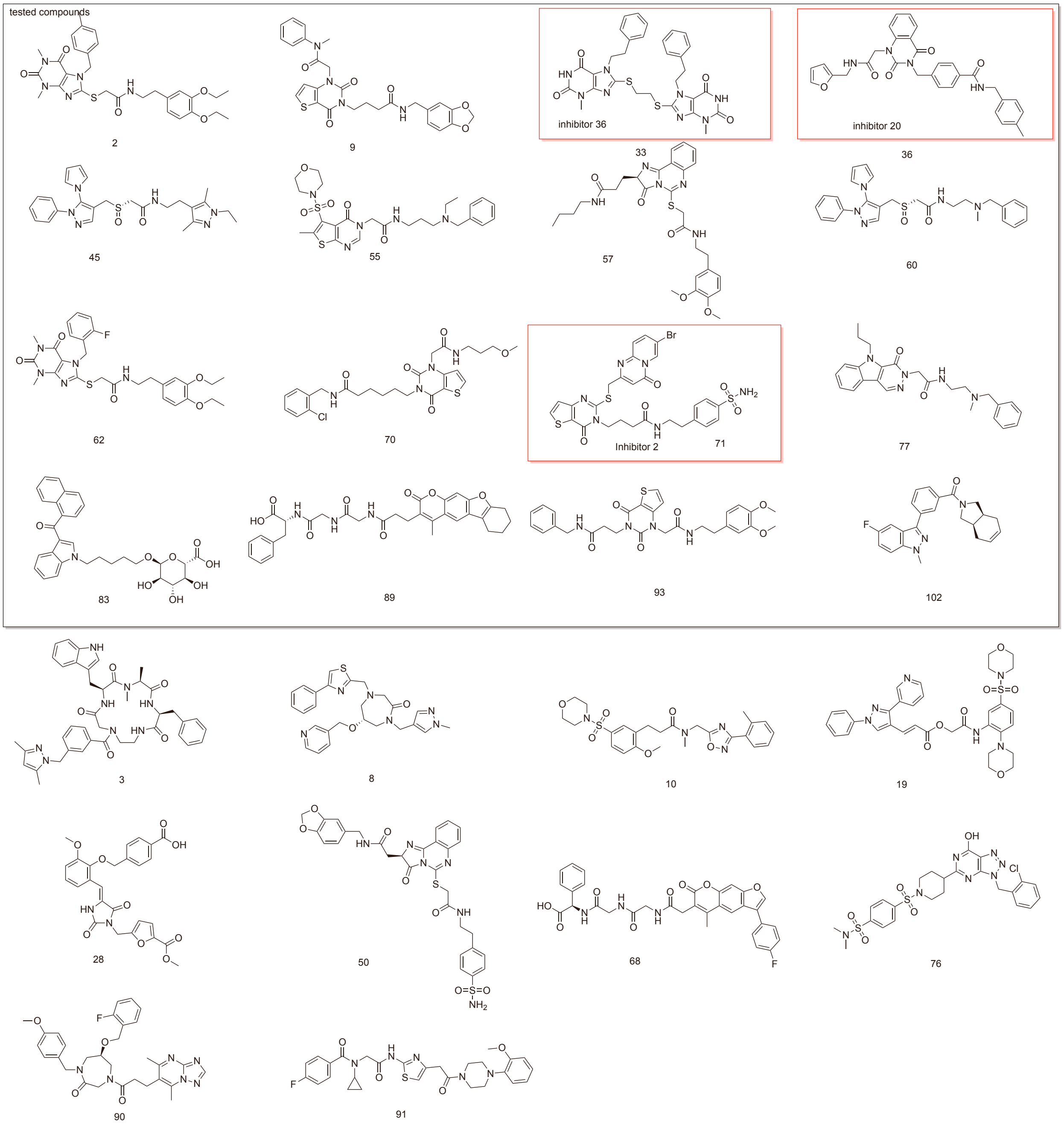

Figure S3. Group C of the top screening results. The boxed ones were purchased for inhibitor screening, the highlighted ones were validated as inhibitors. The docking score rank of each is noted with a number (see Table S2).

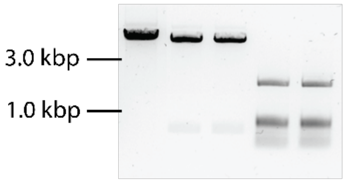

Figure S4. Test of general endonuclease inhibition. Lane 1: Positive control, linearized plasmid. Lanes 2&3: XhoI with and without inhibitor **20.** Lanes 4&5: DpnI with and without inhibitor **20.**

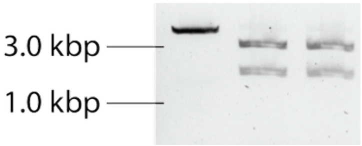

Figure S5. Test of Cas9 nuclease inhibition. Lane 1: Positive control, linearized plasmid treated in reaction condition lacking Cas9 nuclease. Lane 2: linearized plasmid treated in reaction condition including Cas9 nuclease but no inhibitor (DMSO only). Lane 3: linearized plasmid treated in reaction condition including Cas9 nuclease and inhibitor **20** (100 μM).

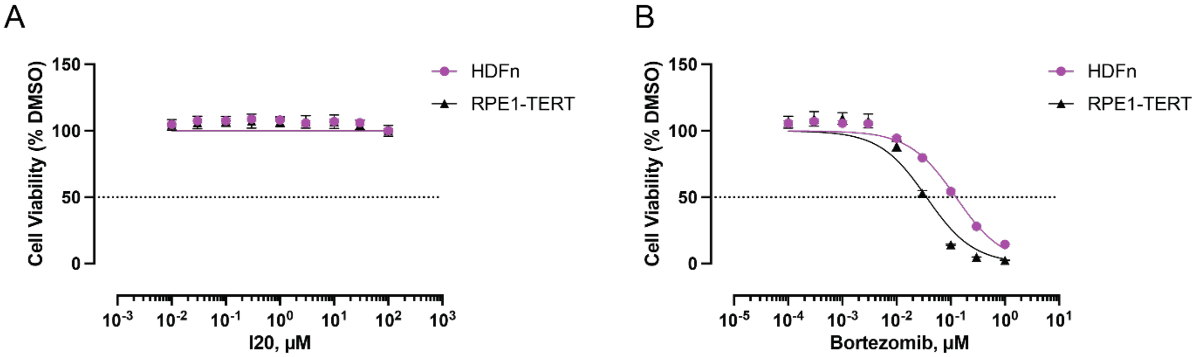

Figure S6. Toxicity test in human cells. Cell viability of two diploid human cell lines (dermal fibroblasts, HDFn; TERT-immortalized retinal pigment epithelial cells, RPE1-TERT), assessed with Promega Cell Titer Glo 2.0. Compound 20 was tested (A), along with Bortezomib as a positive control (B). Data are represented as the average ± SEM from 6 technical replicates. Representative data from one of two independent biological replicates is shown.

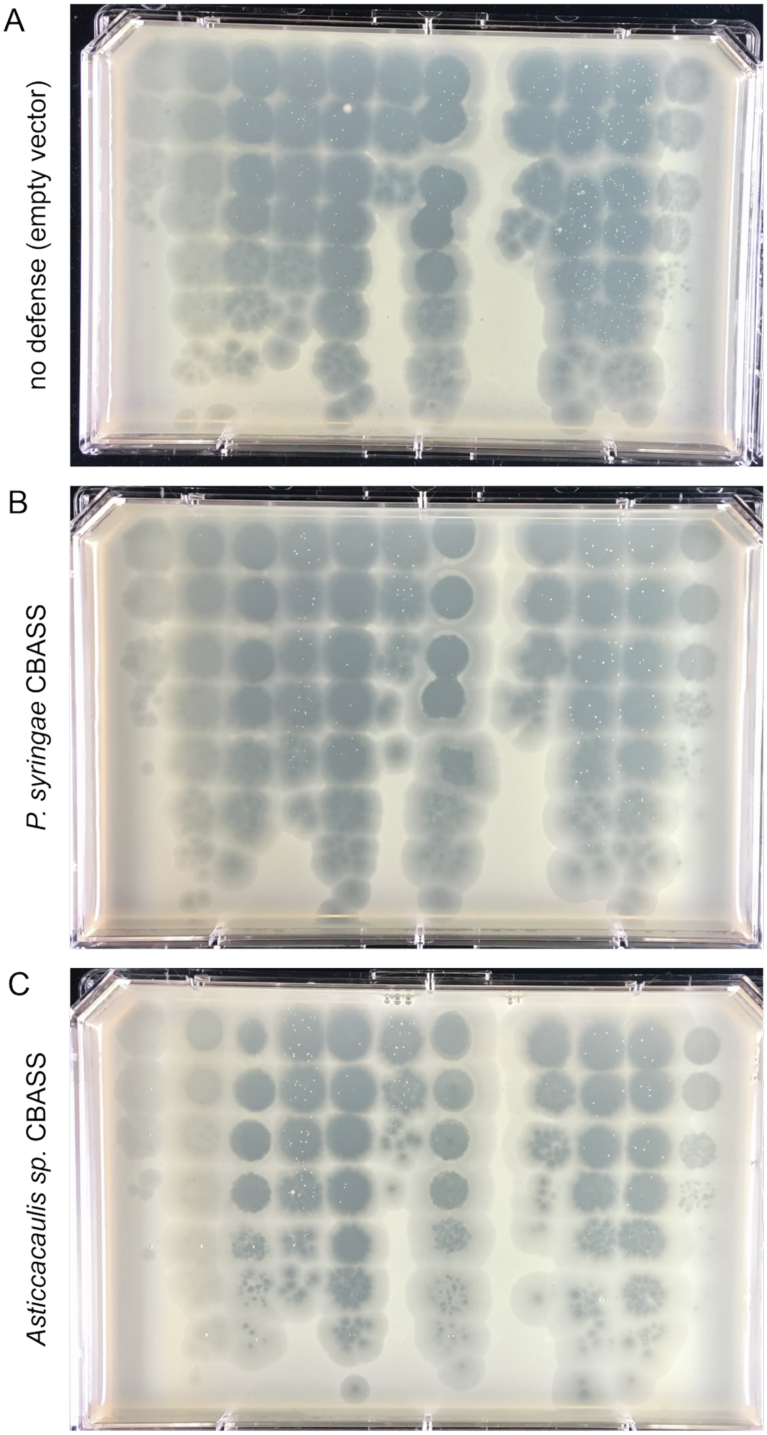

Figure S7. Screen for coliphages targeted by CBASS. BASEL phages 1–12 were tested by plaque assay on *E. coli* expressing no defense (A), the Cap5 CBASS system from *P. syringae* (B), or the Cap5 CBASS system from *Asticcacaulis* sp. (C). 10-fold dilutions of phage lysates were added vertically, and the phages 1–12 were added in sequence horizontally. Phage 8 failed to form plaques, but all others showed no difference between defended and non-defended *E. coli*.

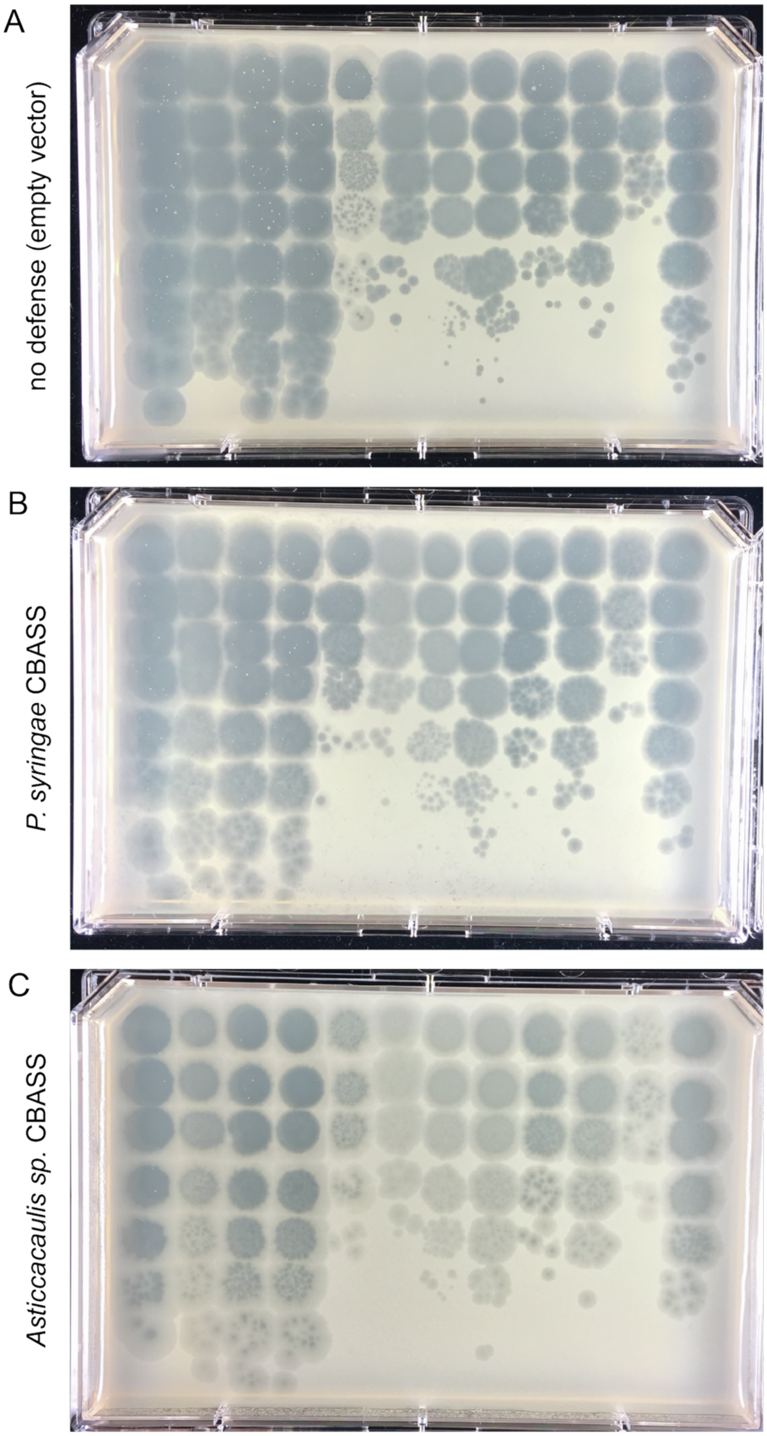

Figure S8. Screen for coliphages targeted by CBASS. BASEL phages 13–24 were tested by plaque assay on *E. coli* expressing no defense (A), the Cap5 CBASS system from *P. syringae* (B), or the Cap5 CBASS system from *Asticcacaulis* sp. (C). 10-fold dilutions of phage lysates were added vertically, and the phages 13–24 were added in sequence horizontally. Phages showed no difference between defended and non-defended *E. coli*.

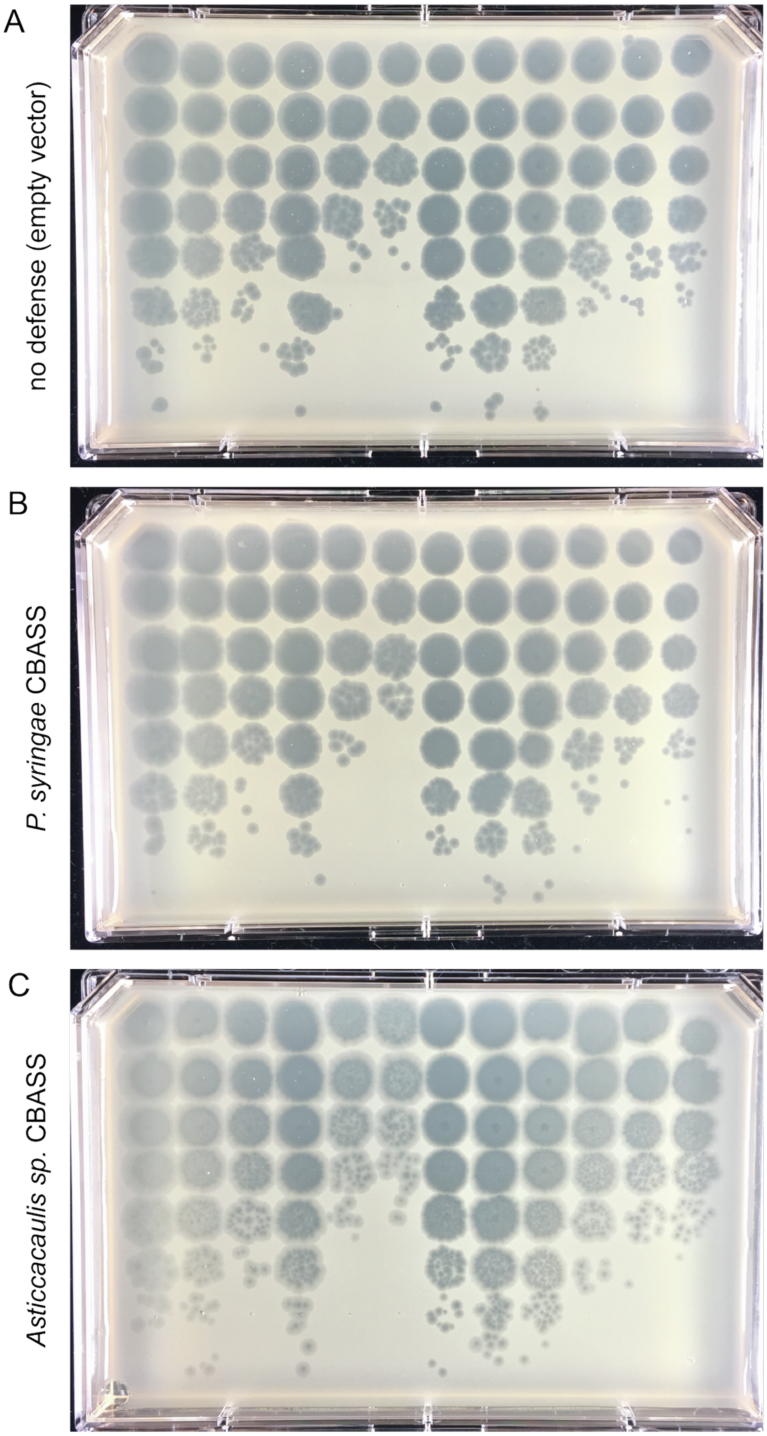

Figure S9. Screen for coliphages targeted by CBASS. BASEL phages 25–36 were tested by plaque assay on *E. coli* expressing no defense (A), the Cap5 CBASS system from *P. syringae* (B), or the Cap5 CBASS system from *Asticcacaulis* sp. (C). 10-fold dilutions of phage lysates were added vertically, and the phages 25–36 were added in sequence horizontally. Phages showed no difference between defended and non-defended *E. coli*.

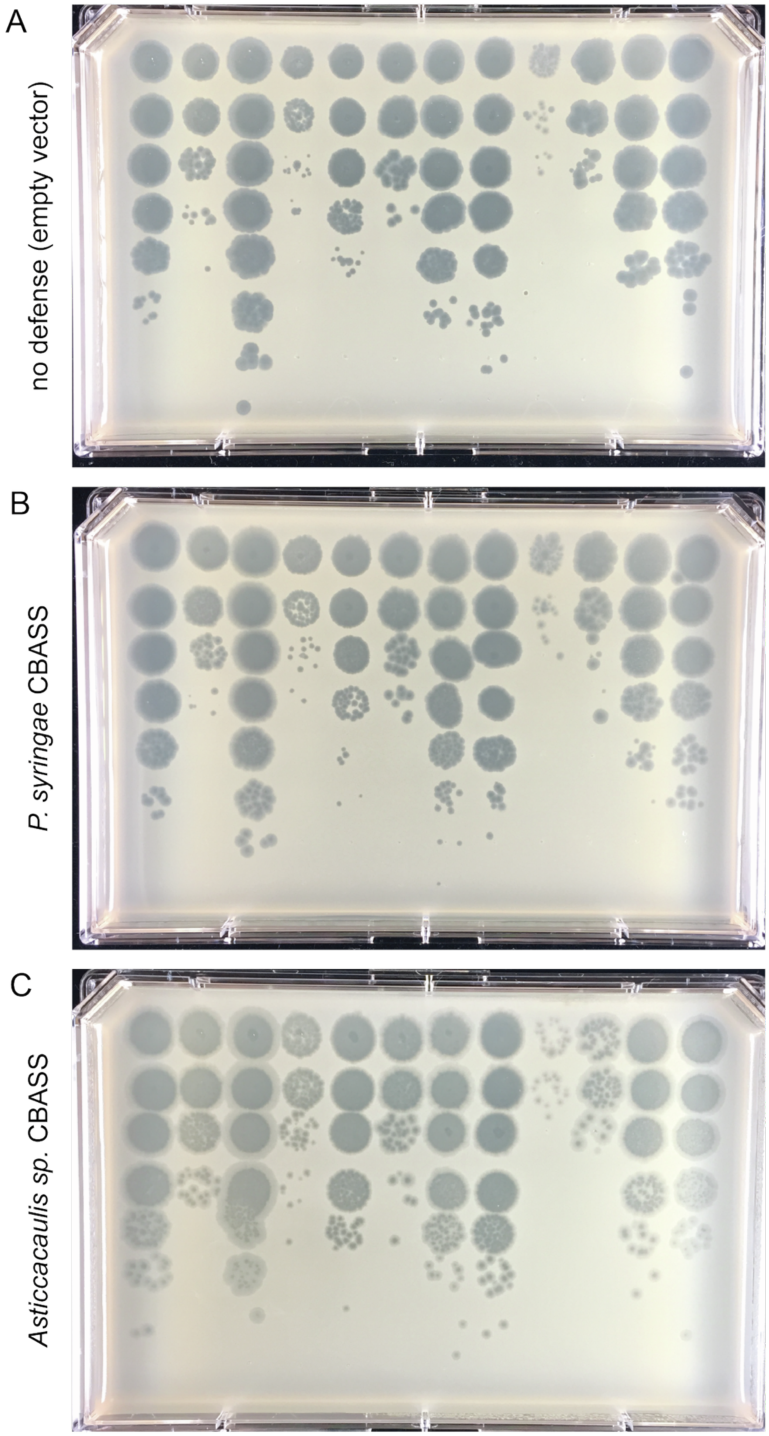

Figure S10. Screen for coliphages targeted by CBASS. BASEL phages 37–48 were tested by plaque assay on *E. coli* expressing no defense (A), the Cap5 CBASS system from *P. syringae* (B), or the Cap5 CBASS system from *Asticcacaulis* sp. (C). 10-fold dilutions of phage lysates were added vertically, and the phages 37–48 were added in sequence horizontally. Phages showed no difference between defended and non-defended *E. coli*.

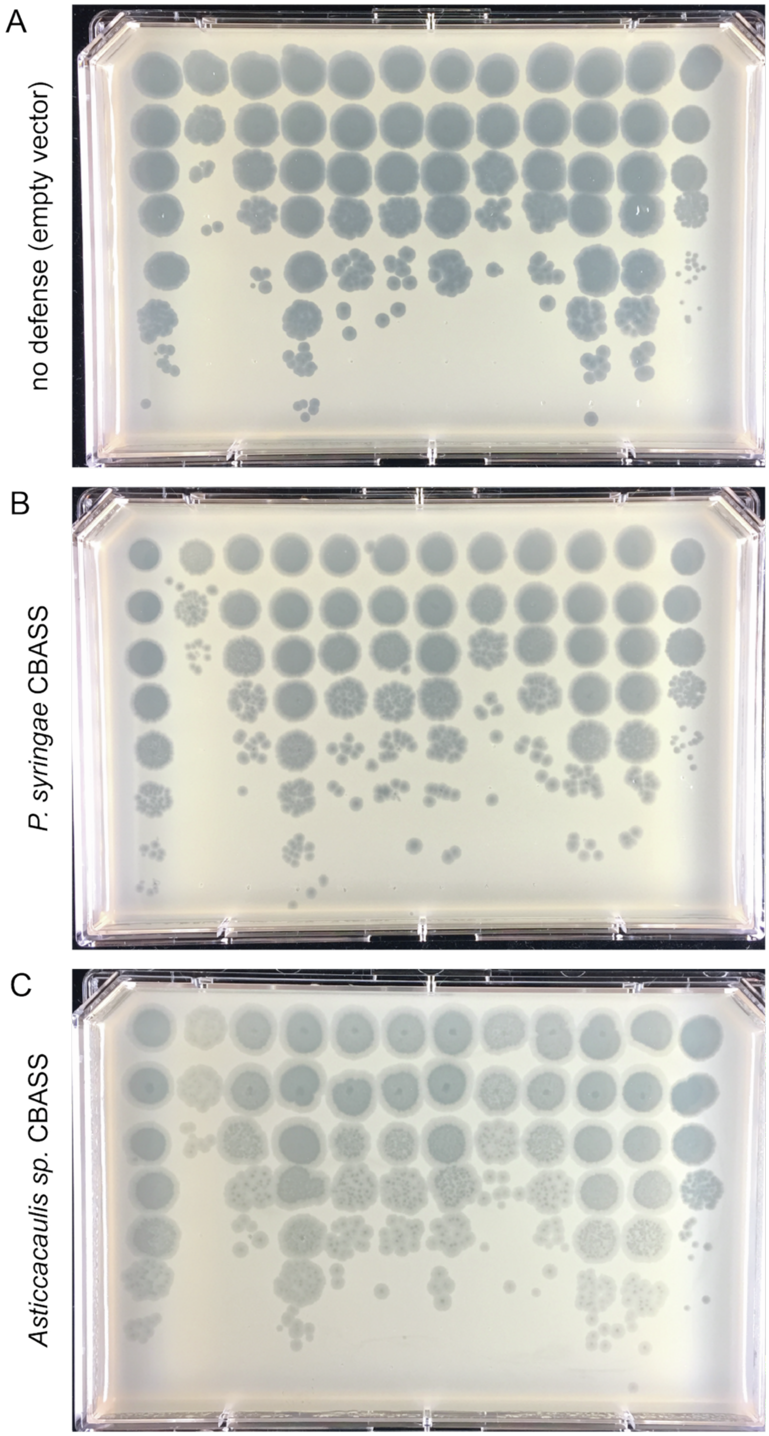

Figure S11. Screen for coliphages targeted by CBASS. BASEL phages 49–60 were tested by plaque assay on *E. coli* expressing no defense (A), the Cap5 CBASS system from *P. syringae* (B), or the Cap5 CBASS system from *Asticcacaulis* sp. (C). 10-fold dilutions of phage lysates were added vertically, and the phages 49–60 were added in sequence horizontally. Phages showed no difference between defended and non-defended *E. coli*.

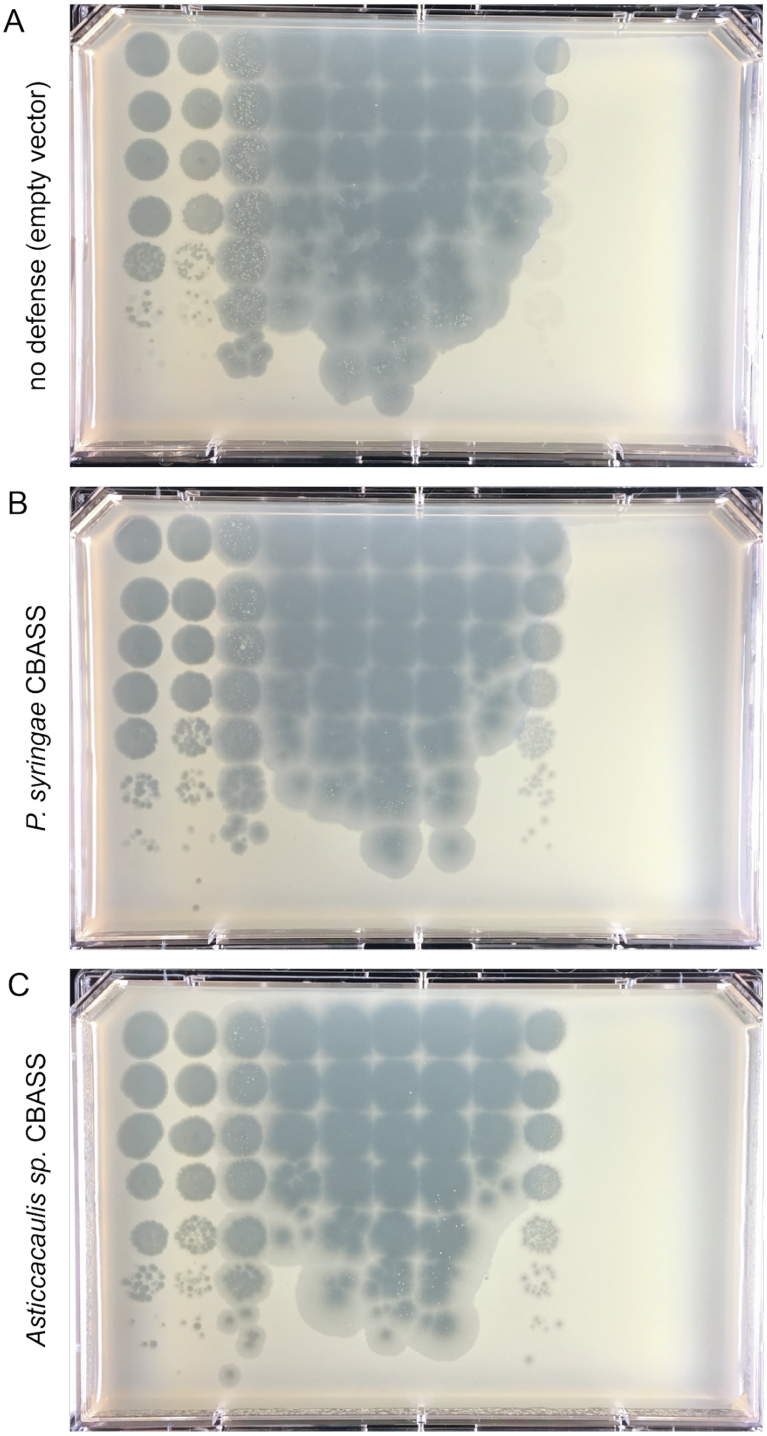

Figure S12. Screen for coliphages targeted by CBASS. BASEL phages 61–69 were tested by plaque assay on *E. coli* expressing no defense (A), the Cap5 CBASS system from *P. syringae* (B), or the Cap5 CBASS system from *Asticcacaulis* sp. (C). 10-fold dilutions of phage lysates were added vertically, and the phages 61–69 were added in sequence horizontally. Phages showed no difference between defended and non-defended *E. coli*.

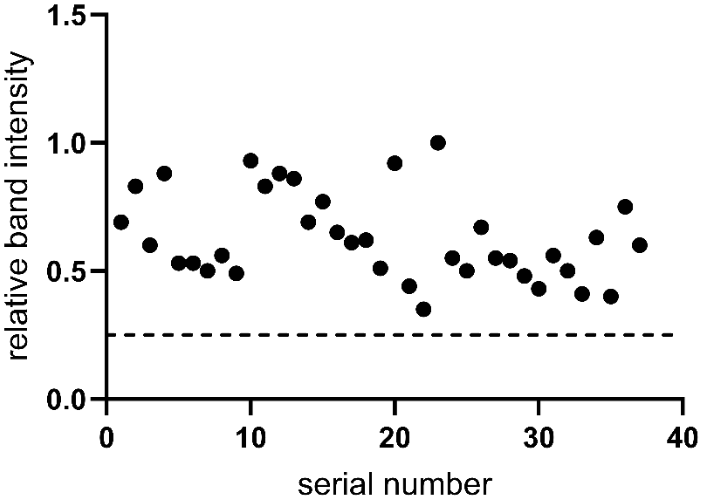

Figure S13. Screen for PsCap5 agonists. No molecules induced substantial DNase activity in the absence of cGAMP.

SUPPLEMENTARY TABLES

Table S1. IC_{50}s of 10 analogs of compound 20.

number	chemdiv number	structure	IC50
1	C260-2750	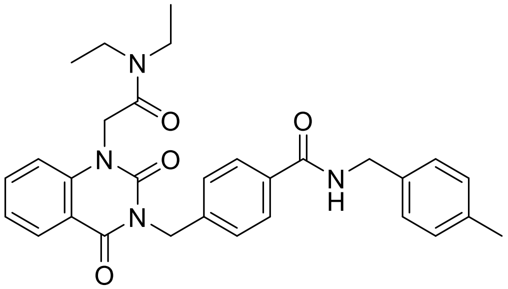	N/A
2	C200-9013	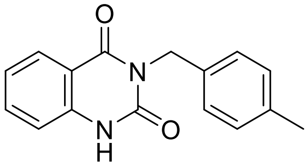	663µM
3	C190-0201	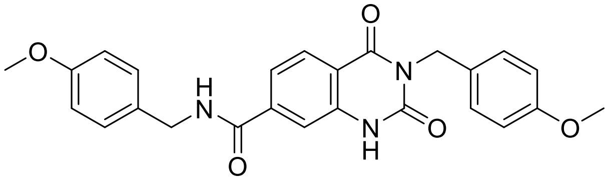	N/A
4	Y700-0269	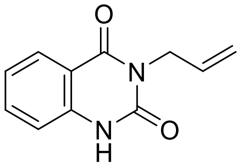	N/A
5	C200-0438	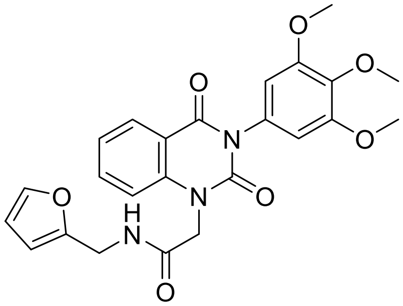	N/A
6	C200-8044	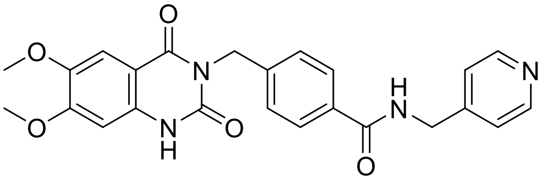	N/A

7	8014-9928	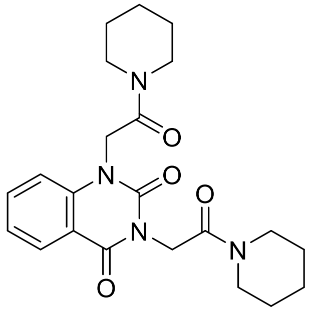	N/A
8	C260-2757	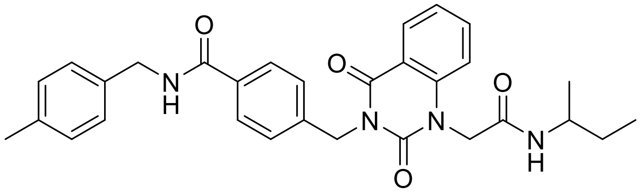	117µM
9	8014-7830	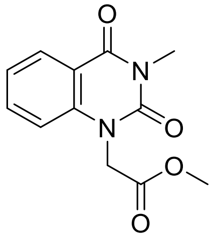	N/A
10	Z606-8908	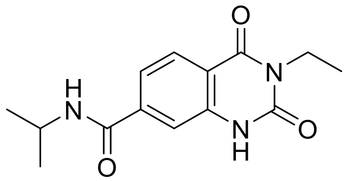	N/A

Table S2. Top 102 scoring compounds from docking.

screening number	ranking number	ZINC number	Cluster	ranking in the initial screening (letter indicates batch, A = smallest molecules, H = largest molecules)
(I3)	1	ZINC000098049432	A	H90
(I26)	2	ZINC000000720462	C	H99
	3	ZINC000952870227	C	H110
	7	ZINC000035376665	B	H145
	8	ZINC000014742824	C	H147
(I13)	9	ZINC000101318681	C	H243
	10	ZINC000225549157	C	H258

	14	ZINC000100852630	A	H52
(I7)	16	ZINC000002975643	A	H61
	17	ZINC000012281127	B	H395
(I10)	18	ZINC000150445454	A	H399
	19	ZINC000150481950	C	H281
	22	ZINC000150512857	B	H542
(I22)	25	ZINC000239305992	B	H358
(I6)	27	ZINC000150429693	A	H793
	28	ZINC000409399692	C	H811
(I8)	32	ZINC000102755438	A	H949
(I36)	33	ZINC000005313219	C	H1393
(I20)	36	ZINC000101332069	C	H1612
	37	ZINC000409194453	B	H1717
(I28)	44	ZINC000003052334	A	H2060
(I9)	45	ZINC000021109893	C	H2170
	46	ZINC000223078696	B	H2246
(S7,I37)	48	ZINC000101134543	B	H2344
	50	ZINC000150425237	C	H3465
(I33)	52	ZINC000100928483	A	H3552
(I18)	54	ZINC000101332011	A	H3329
(I21)	55	ZINC000033357208	C	H3350
(I29)	57	ZINC000008608719	C	H3647
(I17)	60	ZINC000021109929	C	H4345
(I24)	62	ZINC000000718627	C	H4900
(I19)	63	ZINC000027533729	B	H4963
(I1)	65	ZINC000102755808	A	H5265
(I27)	66	ZINC000102448205	B	H5393
(I5)	67	ZINC000004177725	A	H5500
	68	ZINC000070707480	C	H5843
(I14)	70	ZINC000033357756	C	H6832
(I2)	71	ZINC000150390101	C	H7142
	72	ZINC000101210492	B	H7160
	73	ZINC000247393601	B	H7469
	74	ZINC000102023541	B	H8303
	75	ZINC000585269614	B	H8311
	76	ZINC000100446268	C	H8513
(I15)	77	ZINC000009153421	C	H8580
	78	ZINC000012245892	B	H8598
	79	ZINC000103003248	B	H8794
	81	ZINC000224203697	B	H9103
(I11)	82	ZINC000008673675	A	H9424
(I30)	83	ZINC000071789393	C	H9698
(I25)	84	ZINC000070655621	B	H2705

	85	ZINC000004257943	B	H10379
	88	ZINC000014533195	B	H10992
(I23)	89	ZINC000070707577	C	H11140
	90	ZINC000102401085	C	H11273
	91	ZINC000000699403	C	H11387
	92	ZINC000033661437	B	H11627
(I16)	93	ZINC000101318245	C	H11660
	94	ZINC000245333837	B	H11798
(I12)	95	ZINC000102611362	A	H11869
(I35)	97	ZINC000299801611	B	H3070
(I31)	98	ZINC000248246055	B	H2681
(I4)	100	ZINC000150443789	A	H2469
(I32)	101	ZINC000002011307	B	C2964
(I34)	102	ZINC000534660370	C	E5508
